# Special Issue “Advances in the Prevention and Treatment of Ischemic Diseases”

**DOI:** 10.3390/ijms27021029

**Published:** 2026-01-20

**Authors:** Egor Y. Plotnikov

**Affiliations:** 1 A.N. Belozersky Institute of Physico-Chemical Biology, Lomonosov Moscow State University, 119991 Moscow, Russia; plotnikov@belozersky.msu.ru; 2 Institute for Artificial Intelligence, Lomonosov Moscow State University, 119992 Moscow, Russia

Ischemic diseases represent a broad and complex group of clinical pathologies caused by acute or chronic tissue perfusion disorders. The wide range of organs affected by ischemia, along with the diverse causes of ischemia, accounts for the pleiotropy of damage mechanisms and differences in tissue repair potential. Recent research increasingly demonstrates that the outcome of ischemic injury is determined not only by the duration of hypoxia or the severity of post-ischemic stress, but also by the different abilities of cells and tissues to form adaptive responses. These responses include metabolic restructuring, intercellular communication, regulation of inflammation, and controlled activation of anti-apoptotic programs [1,2]. Importantly, restoring blood flow alone does not guarantee tissue healing: even with successful reperfusion, metabolic maladaptation, inflammation, and programmed cell death can persist, determining the long-term outcome of damage. Moreover, oxidative stress is characteristic of the reperfusion period [3]. This Special Issue brings together papers that consider ischemia as a complex network of molecular and cellular events determining both damage and repair, which opens up opportunities for targeted intervention at different levels of biological organization.

A key concept explored in several studies is the idea of induced tolerance to ischemia [4]. It is fundamentally important that damage can not only be treated after an ischemic episode, but it can also be significantly reduced by preconditioning tissue for stress. In the experimental work of Tregub et al. (Contribution 1), it was shown that hypoxia and hypercapnia produce a pronounced neuroprotective effect, with their combination having a synergistic impact. At the molecular level, this effect is mediated by a complex rearrangement of signaling pathways, including NF-kB, HIF-1a, mitochondrial potassium channels, and mechanisms that maintain the integrity of the blood–brain barrier. Notably, in this model, hypercapnia not only enhances hypoxic preconditioning but also limits its excessive activation, creating a protective phenotype that is more economical in terms of energy demand. A similar logic of adaptive restructuring appears in the work (Contribution 2), where a non-pharmacological intervention—time-restricted feeding before kidney ischemia–reperfusion—leads to a marked decrease in inflammation, oxidative stress, and apoptosis without altering caloric intake. In both cases, resistance to ischemia arises from the reconfiguration of fundamental regulatory processes rather than the mechanistic suppression of specific damage pathways.

Understanding such adaptive mechanisms requires detailed analysis of receptor signaling and molecular sensors involved in the ischemic response. In this context, the work of Challa et al. (Contribution 3) on the temporal dynamics of purinergic P2 receptor expression after ischemia and reperfusion is particularly noteworthy. The authors demonstrate that, along with conservatively regulated receptors, some receptors exhibit distinct expression patterns, which can alter the interpretation of experimental data and affect the translational potential of pharmacological agents targeting these receptors. These findings underscore that the molecular architecture of the ischemic response is not universal, and a detailed comparison of both experimental models and target tissues is necessary, especially when developing personalized treatment strategies.

At the cellular level, ischemic damage involves not only the death of neurons or epithelial cells but also an active response from glial and stromal tissue components [5,6]. The work of Varlamova et al. (Contribution 4) demonstrates that selenium nanorods can shift reactive astrocytosis toward the A2 phenotype, which is associated with neuroprotection. Key elements of this effect include Ca2^+^ signaling, PI3K-dependent pathways, and actin-mediated endocytosis of nanoparticles. As with hypercapnic hypoxia, this mechanism highlights that cellular protection in ischemia relies less on passively suppressing cell death and more on activating intrinsic adaptive survival and repair programs. The authors show that reactive astrogliosis, a classic consequence of ischemia, can serve as a target for cytoprotective strategies, and that nanomedicine holds significant potential in this area.

The management of intercellular communication and the regenerative niche is increasingly becoming a central focus in cell therapy research, including in the context of ischemia [7]. The review by Chepeleva (Contribution 5) provides a detailed analysis of the various types of cellular products used in coronary heart disease and convincingly demonstrates that the primary therapeutic effect arises not from the direct replacement of lost cardiomyocytes, but from the paracrine activity of transplanted cells. This conclusion aligns with a broader review by Ma et al. (Contribution 6), which considers mesenchymal stromal cells as universal modulators of inflammation, angiogenesis, and endogenous regeneration in various ischemic conditions. Both reviews agree that further advancement of cell therapy requires the integration of genetic engineering and bioengineering to enhance cell survival, homing, and functional activity. These cells are viewed as exogenous modulators of many endogenous processes rather than as “spare parts” for tissue repair [8].

This idea is experimentally supported by the work of Zheng et al. (Contribution 7), in which adipose mesenchymal cells modified with the monomeric form of CXCL12 exhibit significantly greater neuroprotective and angiogenic effects compared to unmodified cells. CXCR4 blockade completely abolishes these effects, highlighting the direct dependence of the observed changes on this signaling pathway. Thus, this work illustrates the shift from the empirical use of stem and progenitor cells to the development of “smart” biological drugs, whose effectiveness is determined by the precision of molecular design rather than merely the origin of the cellular material.

Understanding the molecular mechanisms of ischemic injury inevitably raises the question of its measurability and predictability [9,10]. In this context, the work of Zaharia et al. (Contribution 8) demonstrates the potential of caspase-3 as a systemic marker of apoptotic cell death in acute ischemic stroke. Although the level of this marker does not correlate directly with clinical severity, its prognostic significance in a subgroup of severe patients indicates a more complex relationship between the intensity of apoptosis and ischemic outcomes. These findings underscore the need to integrate molecular biomarkers into future personalized intervention strategies.

Collectively, the works presented in this Special Issue conceptualize ischemia as a dynamic, complexly regulated process, with outcomes determined by the balance between damage and adaptation. The articles form a logical axis: systemic preconditioning increases tissue resistance to future ischemic events; cytoprotective and pharmacological approaches, including nanomedicine, limit the cascade of damage; cellular products, including genetically modified ones, stimulate regeneration; and molecular biomarkers enable quantification of damage and intervention effectiveness. All these approaches converge on one point: effective prevention and treatment of ischemic diseases are impossible without a deep understanding of the cellular and molecular mechanisms underlying tissue resistance and regeneration. This integration dissolves the rigid boundary between prevention and therapy, transforming them into elements of a unified, molecular-based strategy for managing ischemic damage. This integrated, mechanism-focused approach is reflected in the articles in this Special Issue.

## References

[1] Dirnagl U., Iadecola C., Moskowitz M.A. (1999). Pathobiology of Ischaemic Stroke: An Integrated View. Trends Neurosci..

[2] Hausenloy D.J., Yellon D.M. (2013). Myocardial Ischemia-Reperfusion Injury: A Neglected Therapeutic Target. J. Clin. Investig..

[3] Kalogeris T., Bao Y., Korthuis R.J. (2014). Mitochondrial Reactive Oxygen Species: A Double Edged Sword in Ischemia/Reperfusion vs Preconditioning. Redox Biol..

[4] Kitagawa K., Matsumoto M., Tagaya M., Hata R., Ueda H., Niinobe M., Handa N., Fukunaga R., Kimura K., Mikoshiba K. (1990). ‘Ischemic Tolerance’ Phenomenon Found in the Brain. Brain Res..

[5] Xu S., Lu J., Shao A., Zhang J.H., Zhang J. (2020). Glial Cells: Role of the Immune Response in Ischemic Stroke. Front. Immunol..

[6] Chen Y., Lin L., Tao X., Song Y., Cui J., Wan J. (2019). The Role of Podocyte Damage in the Etiology of Ischemia-Reperfusion Acute Kidney Injury and Post-Injury Fibrosis. BMC Nephrol..

[7] Martins-Marques T., Hausenloy D.J., Sluijter J.P.G., Leybaert L., Girao H. (2021). Intercellular Communication in the Heart: Therapeutic Opportunities for Cardiac Ischemia. Trends Mol. Med..

[8] Wong V.W., Sorkin M., Gurtner G.C. (2013). Enabling Stem Cell Therapies for Tissue Repair: Current and Future Challenges. Biotechnol. Adv..

[9] Bsat S., Halaoui A., Kobeissy F., Moussalem C., El Houshiemy M.N., Kawtharani S., Omeis I. (2021). Acute Ischemic Stroke Biomarkers: A New Era with Diagnostic Promise?. Acute Med. Surg..

[10] Vaidya V.S., Waikar S.S., Ferguson M.A., Collings F.B., Sunderland K., Gioules C., Bradwin G., Matsouaka R., Betensky R.A., Curhan G.C. (2008). Urinary Biomarkers for Sensitive and Specific Detection of Acute Kidney Injury in Humans. Clin. Transl. Sci..

